# A new biologging approach reveals unique flightless molt strategies of Atlantic puffins

**DOI:** 10.1002/ece3.9579

**Published:** 2022-12-13

**Authors:** Jamie Hendrick Darby, Mike P. Harris, Sarah Wanless, John L. Quinn, Vegard Sandøy Bråthen, Annette L. Fayet, Manon Clairbaux, Tom Hart, Tim Guilford, Robin Freeman, Mark John Jessopp

**Affiliations:** ^1^ School of Biological, Environmental and Earth Sciences University College Cork Cork Ireland; ^2^ MaREI Centre for Energy, Climate and Marine, Environmental Research Institute University College Cork Cork Ireland; ^3^ UK Centre for Ecology & Hydrology Penicuik UK; ^4^ Norwegian Institute for Nature Research Trondheim Norway; ^5^ Department of Zoology University of Oxford Oxford UK; ^6^ Institute of Zoology Zoological Society of London London UK

**Keywords:** auks, flightless moult, flightless molt, *Fratercula*, Geolocator tracking, life‐history strategies, puffin, seabird ecology, wet–dry sensor

## Abstract

Animal‐borne telemetry devices provide essential insights into the life‐history strategies of far‐ranging species and allow us to understand how they interact with their environment. Many species in the seabird family *Alcidae* undergo a synchronous molt of all primary flight feathers during the non‐breeding season, making them flightless and more susceptible to environmental stressors, including severe storms and prey shortages. However, the timing and location of molt remain largely unknown, with most information coming from studies on birds killed by storms or shot by hunters for food. Using light‐level geolocators with saltwater immersion loggers, we develop a method for determining flightless periods in the context of the annual cycle. Four Atlantic puffins (*Fratercula arctica*) were equipped with geolocator/immersion loggers on each leg to attempt to overcome issues of leg tucking in plumage while sitting on the water, which confounds the interpretation of logger data. Light‐level and saltwater immersion time‐series data were combined to correct for this issue. This approach was adapted and applied to 40 puffins equipped with the standard practice deployments of geolocators on one leg only. Flightless periods consistent with molt were identified in the dual‐equipped birds, whereas molt identification in single‐equipped birds was less effective and definitive and should be treated with caution. Within the dual‐equipped sample, we present evidence for two flightless molt periods per non‐breeding season in two puffins that undertook more extensive migrations (>2000 km) and were flightless for up to 77 days in a single non‐breeding season. A biannual flight feather molt is highly unusual among non‐passerine birds and may be unique to birds that undergo catastrophic molt, i.e., become flightless when molting. Although our conclusions are based on a small sample, we have established a freely available methodological framework for future investigation of the molt patterns of this and other seabird species.

## Graphical abstract

Atlantic puffins are flightless while they molt and regrow their flight feathers. We have developed a new method that uses information from biologgers to investigate where and when this occurs.

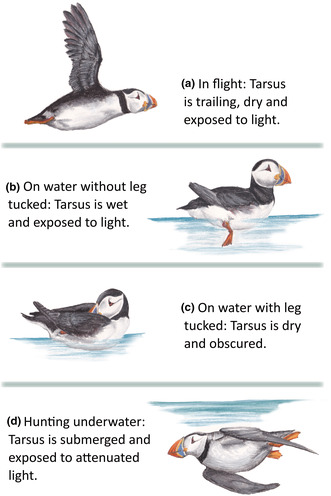

## 1 INTRODUCTION

Biologging and telemetry studies have greatly advanced our knowledge of the behavior and distribution of far‐ranging animal species (e.g., Jouventin & Weimerskirch, 1990; Kooyman, 1966). They have also provided insights into their behavior, especially when direct observation is impossible or impractical (e.g., Michel et al., 2022; Wilson et al., 1991), which is often the case for the many marine species that spend prolonged periods at sea far from land (e.g., Bennison et al., 2019; Brooke, 2018; Doyle et al., 2015; Weimerskirch et al., 2006). Using telemetry, the behavior of far‐ranging species can be defined at a relatively fine temporal scale, such as diel patterns of movement (Seyer et al., 2021), and over longer time series to describe life‐history strategies, such as migration (Amélineau et al., 2021) or periodic molt (Grissot et al., 2020). By looking at the behavior of an animal in relation to its spatial and temporal distribution, it is possible to identify key areas of conservation concern and identify drivers of declines (Fayet et al., 2021; Frederiksen et al., 2012).

Many of the world's seabirds are threatened and declining (Dias et al., 2019; Paleczny et al., 2015), creating a pressing need to better understand the vulnerable stages of their annual cycle. Seabirds tend to be highly susceptible to the impacts of climate change, including sea temperature rise and shifts in prey distribution and abundance (Durant et al., 2003; Sandvik et al., 2005), as well as extreme weather events (Clairbaux et al., 2021), with large wrecks recorded following severe winter storms at sea (Anker‐Nilssen et al., 2018; Harris et al., 2014; Morley et al., 2017). Larger members of the seabird family *Alcidae* (hereafter alcids) molt all their primary flight feathers simultaneously, or at least within a few days (Peery et al., 2008; Thompson et al., 1998), leading to a protracted flightless period, placing them at greater risk from such dynamic stressors. Alcids are often the most common species washed ashore in storm wrecks in the northern hemisphere (Morley et al., 2017). Obligatory flightless molt places birds at greater risk from storm events, since they are unable to fly to avoid the storm track. Being flightless also potentially reduces alcids' ability to escape from marine predators (Ulman et al., 2015) and increases their vulnerability to surface pollutants because of the increased time spent on the water surface and the inability to escape expansive films of harmful substances such as petroleum oil (Robertson et al., 2012).

The Atlantic puffin (*Fratercula arctica*), hereafter puffin, is an alcid species that has undergone rapid population declines across most of its European breeding range during the 2000s (Harris & Wanless, 2011), leading to its classification as Endangered in Europe by the European Red List Assessment in 2015 (BirdLife International, 2015). Because puffins become flightless during molt (Gaston & Jones, 1998; Harris et al., 2014), they must carefully time and locate their molt to coincide with sufficient food availability, which can be patchily distributed at sea (Fauchald, 2009; Jessopp et al., 2013). Clairbaux et al. (2021) calculated the fasting endurance of puffins as 6.5 (±2.5) days in mid‐autumn and 4.6 (±0.6) days in winter. Local depletion of food during molt puts puffins at risk of starvation. Anker‐Nilssen et al. (2018) found that most puffins washed ashore in a post‐storm wreck in southwest Norway in early 2016 were in the late stage of primary molt, and almost all individuals were emaciated. Molt may have prevented them from escaping the storm when they were flightless, during which they clearly struggled to find food. Similar to other diving seabirds, puffins' feeding strategy and functional dive depth may vary depending on environmental conditions (Darby et al., 2022), which would help to explain their highly varied diet (Baillie & Jones, 2004), especially during the winter, when adverse weather conditions are most likely to impact their foraging behavior (Falk et al., 1992; Harris, Leopold, et al., 2015).

**FIGURE 1:**
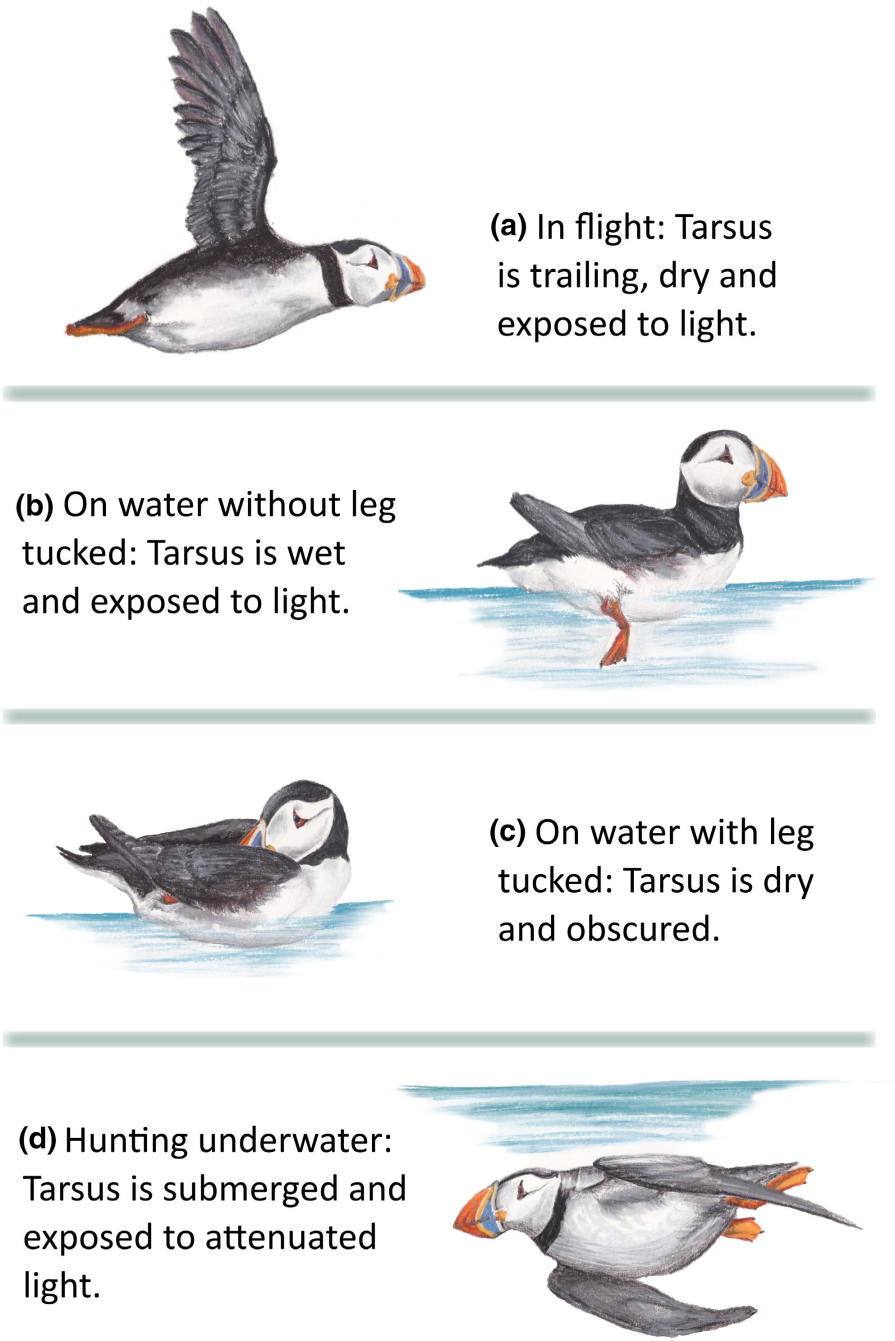
Different puffins' at‐sea behaviors and the position of the tarsus during each. Loggers were mounted on the tarsus of study individuals. Puffins trail their tarsus in flight (a), which leaves the geolocator exposed to light and dry. Puffins sitting on the surface of the water may lower one or both legs for paddling and balance (b), which leaves the tarsus submerged and exposed to light. Puffins inactive on the water may also tuck one leg into their plumage (c), obscuring the tarsus from daylight and keeping it dry. Puffins hunting for prey underwater will have their tarsus submerged and exposed to light levels attenuated by water (d), although this is unlikely to reduce actual light readings, which are taken as a maximum over 5‐ or 10‐ min intervals.

The duration, timing, and location of molt in puffins are major gaps in our knowledge of this species because molt occurs during the non‐breeding season when birds are at sea, often far away from their colonies (Fayet et al., 2017). Harris et al. (2014, 2022) assessed molt stage based on feather development in a large sample of puffins either washed ashore during storm wrecks or shot by hunters for food. These studies found that molt of all primary flight feathers occurred at any time from September to March, with peaks in October and March. This variable timing contrasts with other alcids, whose molt typically occurs shortly after the breeding season (Gaston & Jones, 1998; Peery et al., 2008). Identifying the flightless period of puffins using biologging studies has also proven difficult. Leg‐mounted saltwater immersion loggers are commonly used to classify seabird behavior during the non‐breeding season. Reduced time spent flying during flight feather molt, for instance, is usually reflected by an elevated proportion of time the leg and logger are wet (Grissot et al., 2020). Puffins, like other alcids, repeatedly tuck their legs into the plumage when on the water (Harris et al., 2010; Linnebjerg et al., 2014; I. Sempere, Oceanário de Lisboa, pers. comms, Figure 1), confounding simple behavior classification using these loggers.

This study uses light‐level geolocators with integrated saltwater immersion switches deployed on puffins to identify patterns of behavior thought to be consistent with flightless molt. By combining data from four individuals with a geolocator on each leg (dual‐equipped birds), we developed a behavioral classification method using raw light and saltwater immersion data. We show that we can use results from dual‐equipped birds to quantify and correct for behaviors that would confound traditional methods, and enable us to identify flightless periods assumed to represent molt. We then adapt and validate this method for single‐equipped birds, for which there are far more data. This approach may help us to identify overwintering strategies and areas of conservation concern for puffins and other alcids, whose highly restricted mobility during flightless molt may exacerbate the negative impacts of environmental threats (Ausems et al., 2021).

## 2 METHODS

### 2.1 Deployment and recovery of geolocator devices

Geolocators (seven BAS Mk18‐L and one Mk14) were deployed on both legs of four adult puffins during the 2010 summer breeding season on Skomer Island, Wales (51.737 N, 5.297 W). Single geolocators (31 Biotrack [2012]/Lotek [2020] Mk4083 and 12 BAS Mk18 [2010]) were deployed on 40 adult puffins during the breeding season in 2010, 2012, and 2020 on Skellig Michael, Ireland (51.771 N, 10.539 W). Birds at both colonies were captured during chick rearing either using purse nets at burrow entrances or by hand from the burrow and weighed and fitted with geolocators before being released back to their burrow. Capture and handling times were kept to a minimum. Geolocators were attached to a colored plastic ring fitted around the tarsus, with total deployment weight (devices plus leg ring and cable tie attachment) always <2% body mass, under 5 g for dual‐equipped loggers and under 3 g for single‐equipped loggers. Device effects were carefully monitored, especially for the dual‐equipped individuals, as this method is unorthodox and may impact birds to a greater degree than single tags. According to the light data recorded by the tags, dual‐equipped birds continued to return to their burrows, presumably to provision chicks, after the tags were attached. All four returned to the colony the following year, with three confirmed as breeding and one not confirmed only due to the inaccessibility of its nest chamber. The overwintering areas for the dual‐equipped birds were representative of the areas used by birds with single tags equipped (Fayet et al., 2016).

The attached geolocators measured light every minute in 6‐bit units from 0 (light is below civil twilight; sun >~6 degrees below the horizon) to 64 (the sun is well above the horizon) and saved the maximum light level sampled in 5‐min intervals or 10‐min intervals for the single Mk14 logger used. Saltwater immersion data were sampled every 3 s as binary units of 0 (dry) or 1 (wet) and the number of wet samples in 10‐min intervals were saved as values between 0 (all dry) and 200 (all wet). Devices were recovered from birds during the subsequent breeding seasons. All work was carried out under license from the British Trust for Ornithology (CO/6143, C/5311), with work in Ireland further licensed by the National Parks and Wildlife Service (06/2020, C41/2020, 26/2010, C051/2011, C116/2012, C039/2013, 11/2013). The attachment of dual‐equipped geolocators was granted ethical approval by the British Trust for Ornithology Unconventional Methods Technical Panel, with ethical approval for handling and tagging in Ireland also approved by the University College Cork Animal Ethics Committee. All analyses were performed using R version 4.1.2 (R Core Team, 2021), and all code to run these analyses is available online (github.com/JamieHDarby/puffin_moult).

### 2.2 Location data

Positions were obtained from light‐level data using a threshold method following established procedures (Lisovski et al., 2019). Twilight events were identified when raw light level crossed a threshold of 1 which separates day from night. From these twilight events, latitude can be calculated using the length of day and night, and longitudes based on the time of noon and midnight. Twilight events were validated using the *twilightEdit* function from the *TwGeos* package (Lisovski et al., 2016), which utilizes a moving window approach to recognizing improbable twilight events by comparing twilight times across multiple days. This function either adjusts or deletes individual anomalous twilights due to prolonged leg tucking, for instance, depending on their incongruity within the time series. Sun angle calibration for calculating latitudes was performed using the Hills–Ekstrom algorithm for identifying the most likely solar zenith based on multiple runs of latitude prediction. This was implemented using the *SGAT* package (Sumner et al., 2009). Around equinox events, the precision of latitude estimates drops considerably as the difference in day length gets less distinct across the latitudinal gradient. Latitudes were therefore smoothed using locally weighted smoothing (LOESS) for a 2‐month period around the equinox in autumn (Aug 22 to Oct 23) and spring (Feb 19 to Apr 22), then smoothed again using linear interpolation for a subsequence of dates closer to the equinoxes (Sep 1 to Oct 13 and Mar 1 to Apr 12). Areas of apparent residency were identified over the non‐breeding season (August to March) using Lavielle segmentation, following methods from Amélineau et al. (2021), and the mean distance to the colony of each area of residency was calculated. The migratory effort was described as the distance to the furthest point of residency from the colony.

### 2.3 Accounting for leg‐tucking behavior

Puffins tend to tuck their legs into their plumage while resting on the water, usually just one at a time, but sometimes both (Figure S1, pers. obs.), leaving the logger dry despite the puffin being on the water surface (Figure 1). This means it can be difficult to distinguish between flight and rest from a geolocator immersion signal alone (Fayet et al., 2016). We developed a new method that identified and accounted for leg tucking using concurrent light signals. Raw light readings were scaled from 0 to 1. We calculated the expected solar angle for each data point (angle between the sun and horizon) based on time, date, and location using the *oce* package (Kelley & Richards, 2020). We then modeled the scaled geolocator light reading against solar angle in a generalized additive model (GAM) using the *gam* function of the *mgcv* package (Wood, 2008) over a subset of 100,000 light data points. Using this model, we predicted expected light readings for all data points based on the solar angle at the time and position of the fix. If light readings were anomalously low, >2× standard deviation (SD) below the predicted value, it was assumed that the bird was exposed to higher light levels but was leg tucking for the duration of that fix interval, obscuring the logger. Fixes on either side of a light‐informed tucking event were also classified as tucking, given that when puffins initially tuck or untuck their legs, maximum light levels in that fix would not be anomalously low, but the proportion of time the tag spent immersed would still underestimate the time the puffin spent on the water.

These lower‐than‐expected light fixes were classified as tucking and were appended to concurrent immersion data using a time‐series merge implemented using the *xts* package (Ryan & Ulrich, 2020) to account for missing or delayed points in either data stream. The immersion data points associated with these fixes were adjusted to 100% wet to reflect that they were assumed to be resting on the water despite the logger reading fully or partially dry. This correction could only be applied to data occurring during daylight hours (solar angle >−6 degrees) and not during the hours of darkness.

### 2.4 Combining data from dual‐equipped loggers

Immersion data from both leg tags were combined for each dual‐equipped puffin, again using a time‐series merge implemented using the *xts* package. This time‐series merge accounts for both missing data points and differences in start times between paired loggers, merging the data from one logger to the nearest possible timestamp in the other logger's data stream. Proportions of time spent wet were compared pairwise for each fix interval and the higher value was retained in a single data stream. This meant that even if one leg recorded dry and the other recorded wet for the same time interval, the “wet fix” was preferentially retained. If one leg was submerged, then the puffin must have been resting on the water, and the “dry fix” was an instance of leg tucking not captured by methods described in the previous section. This method may slightly overestimate the time immersed. However, any time spent immersed at all cannot represent directed sustained flight, so this should not have an impact on our results. The time‐series merge we used also accounted for differences in light recording intervals for one individual (EL60648) with different logger types. The data stream with the shorter recording interval was used as the basis for the time‐series merge, and the coarser‐resolution data from the other logger were used to correct the closest data points time‐wise. This mismatch may impact the efficacy of the correction, although no issues were evident when comparing results between this individual and the others.

### 2.5 Identifying molt periods in dual‐equipped birds

We then assumed the corrected proportion of time spent dry per day was the time in flight (prop_flight_) and calculated the 5‐day rolling average of this (prop_flight‐5_) centered on a focal day and calculated using data from 2 days before to 2 days after. This rolling average was used to smooth noise in the data, which was likely caused by leg tucking that our corrections could not detect, and to facilitate the identification of molt periods. Only sections of days with a predicted solar angle of >−3 degrees were used to calculate this proportion, as some anomalous sustained dry periods were retained in nocturnal data points. Although these periods could in fact be due to sustained flight occurring at night, it is more likely due to puffins tucking both legs at once (Robertson et al., 2012, pers. obs.) or visits to the nest burrow approaching the breeding season. The tucking correction applied earlier will not have captured this on either leg's logger, as the expected light level at lower solar angles (<−6 degrees) is zero.

An inferred flightless period was identified as a persistently low set of values of prop_flight‐5_, identified using an incrementing threshold. This threshold value was iteratively increased from 0 by 0.0002 increments until a minimum sequence of 30 consecutive days were defined as below this threshold. Days with prop_flight‐5_ below this threshold value were defined as flightless. This 30‐day minimum sequence of flightless days required to constitute molt was defined according to previous estimates of this species (Harris et al., 2010) and observations of puffins in an aquarium setting (D. Dial, National Aquarium USA, pers. comms). A maximum threshold value of 1% prop_flight‐5_ was applied to restrict the likelihood of falsely identifying molt during periods of reduced flight, during which the puffin was not obligatorily flightless but rather had reduced flying time, likely due to remaining resident in an area of favorable feeding or weather conditions. This meant that the 5‐day rolling average of corrected flight, or prop_flight‐5_, had to remain below 1% for a period to be considered as molt. Molt was therefore defined as a continuous period of at least 30 days, during which little to no flight (<1% of daylight hours) was inferred to have occurred by the processed immersion data. The same process was repeated, omitting any initially identified molt, to explore the possibility of a second flightless molt.

### 2.6 Testing single‐leg data in dual‐equipped birds

A similar molt identification method was attempted using data from each dual‐equipped logger in isolation. As before, the proportion of time spent wet was corrected for tucking behavior using raw light signals. Any points with a solar angle below −3 degrees were omitted for this analysis to ensure that leg tucking was sufficiently captured using light data. Prop_flight_ and prop_flight‐5_ were again calculated, and the same incrementing threshold method was used to identify putative molt periods of a minimum of 30 days duration. A maximum threshold value of 1% prop_flight‐5_ was again applied to restrict the likelihood of falsely identifying molt from noisy immersion time series. Independently derived inferred molt periods were compared to combined data from dual‐equipped loggers. Based on the limited but good agreement (see Section 3), the process was then applied to the geolocator data collected from 40 individuals equipped with single loggers from Skellig Michael, Ireland.

### 2.7 Observations from captive puffins

Several aquaria house puffins as part of displays. Aquarists from four of these facilities provided observations about molting habits of these puffins in an aquarium setting to contextualize results and check whether our conclusions were physiologically viable. We spoke to aquarists from Tierpark Bern, Switzerland, the National Aquarium, USA, Oceanário de Lisboa, Portugal, and Biodôme de Montréal, Canada. All mentioned a variation in the molting behavior. Meret Huwiler of Tierpark Bern described how juvenile puffins molt their primaries twice in 1 year. Older age classes have a single primary molt, the timing of which advances as they age, from mid‐winter to early autumn. Debra Dial of the National Aquarium described variation in the timing of molt between wild‐caught and captive‐reared individuals initially and highlighted how age, lighting, and the birds' condition can all affect the timing and duration of molt, with the latter estimated as 45–50 days. Ana Ferreira and Irene Sempere of Oceanário de Lisboa described how appetite increases prior to molt, and that puffins do not rest or tuck their legs any more or less during molt than at other stages. All four facilities were able to confirm that puffins tucked their legs into their plumage. Further relevant observations are referenced as personal communications throughout the text.

## 3 RESULTS

### 3.1 Accounting for leg‐tucking behavior

**FIGURE 2:**
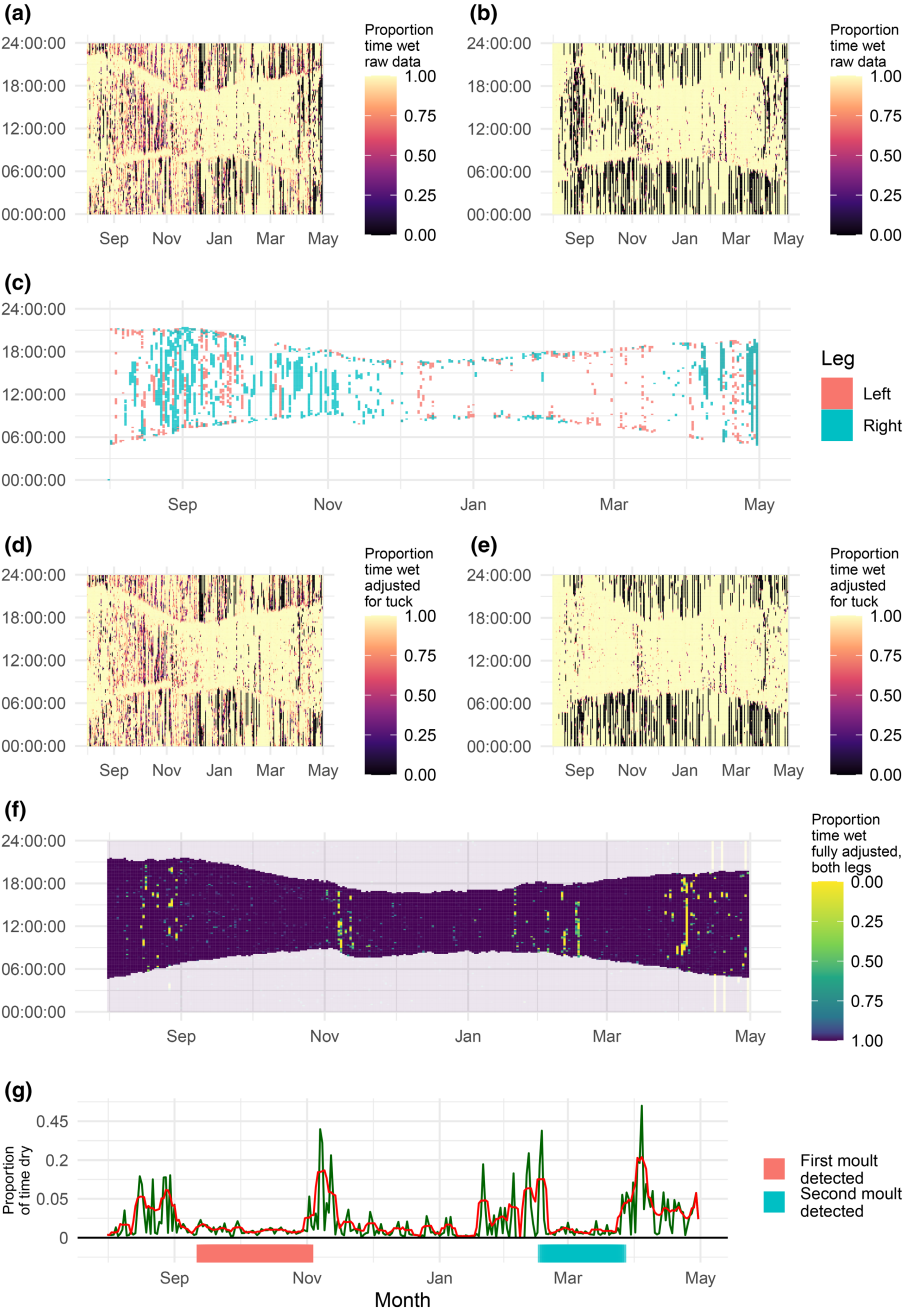
Geolocator data process for a dual‐equipped puffin (EJ47625) for molt identification, accounting for leg tucking using raw light signals, and combining data from loggers on each leg. All data are displayed as a 2D rasterized time series. The *x*‐axis represents the calendar date, and the *y*‐axis represents time in Universal Time Zone. (a, b) The raw saltwater immersion signals from the puffin's left and right leg, respectively. (c) Highlights the data points inferred as leg tucking using the raw light signals. (d, e) The immersion data corrected for leg‐tucking events shown in ‘c’, again for the puffin's left and right leg‐mounted loggers, respectively. (f) The combined minimum of ‘d’ and ‘e’, which represents the most accurate estimate of flight activity, given the data available. In ‘f’, data with a predicted solar angle of <−3 are made semi‐transparent to highlight the time series retained for molt detection. Consistent 0 values at night in ‘f’ during April probably represent time spent in the burrow, i.e., neither leg is wet. (g) A time series of prop_flight_ (green) and prop_flight‐5_ (red). The red bar underneath the plot represents molt inferred on the first iteration of the molt identification process, and the blue bar represents a potential second flightless molt identified by the second iteration. The *y*‐axis of ‘g’ is the square root transformed to facilitate the visualization of positively skewed data, with the actual untransformed values displayed.

Leg tucking was identified using raw light and immersion data streams from loggers from both single‐ and dual‐equipped birds. Clear differences in raw immersion data can be seen from each leg of a single puffin, largely down to leg tucking (Figure 2a,b). Although we capture and account for much of the daylight leg tucking using concurrent light data from the same logger (Figure 2c), differences between corrected immersion time series from each leg suggest that not all leg tucking is accounted for in this method (Figure 2d,e, Table 1). The proportion of time spent dry per day is reduced by >50% when concurrent light levels are used to correct leg tucking, and this corrected proportion is further reduced by >50% when data from two tags are combined (Table 1). Data from both legs are therefore likely necessary to accurately identify flightless periods, i.e., primary feather molt (Figure 2f). Using the dual‐equipped loggers, we calculated that puffins spent 21.7% (SD = 20.1) of daylight hours (solar angle >−3) tucking either or both legs, 10 times greater than the time spent in flight per day (1.9%, SD = 5.8). Puffins spent less than 1% of daylight hours in flight for 75% of days during the non‐breeding period. Although the small sample size precluded detailed analysis, the proportion of time spent leg tucking also varied with individual, time of year, and leg (Appendix S1).

**TABLE 1:** Time spent dry according to raw immersion signal (no adjustment), immersion data corrected for leg tucking using concurrent light data only (light adjustment), and immersion data corrected using corresponding data from another logger on the same bird (dual tag).

	No adjustment (%)	Light adjustment (%)	Dual tag (%)
Time spent dry/day	11.8 ± 14.4	5.3 ± 8.5	1.9 ± 5.8
Time spent dry/NBS	11.8 ± 5.1	5.3 ± 1.9	1.9 ± 0.8
>95% dry fixes/NBS	6.6 ± 3.78	1.6 ± 1.0	0.8 ± 0.6

*Note*: Behaviors presented are time spent dry per day, time spent dry per non‐breeding season per individual (NBS), and the proportion of data points that were recorded as being >95% dry per NBS. Values are means ± SD. All values are calculated as proportions of daylight hours (solar angle >−3).

### 3.2 Identifying molt periods from dual‐deployment birds

**FIGURE 3:**
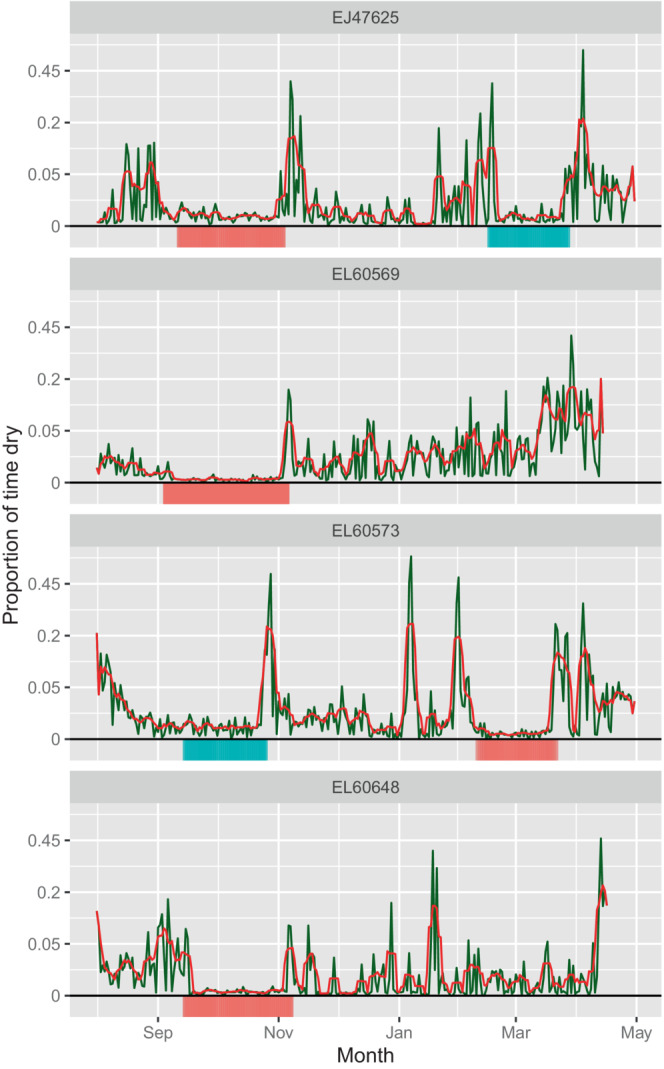
Inferred timing of flightless molt in four dual‐equipped puffins. Immersion data were combined between two geolocators for each puffin. The green line represents time spent dry per day (prop_flight_) and the red line is the 5‐day rolling average of this (prop_flight‐5_). The *y*‐axis is square root transformed, with the actual untransformed values displayed. The red bar underneath each plot represents molt inferred on the first iteration of the molt identification process, and the blue bar represents a potential second flightless molt identified by the second iteration.

Each dual‐equipped puffin had a molt period inferred to begin in September, while two had a second inferred molt period beginning in mid‐February (Table 2 and Figure 3). There was some variation in the duration of molt, from 32 to 63 days. During inferred molt periods, the percentage of time spent dry according to raw immersion signals was 12.8%, while it was reduced to 3.9% when adjusted for leg tucking using data from a single leg. When data streams from both legs were corrected for tucking and combined, the proportion of time spent dry during inferred molt was 0.1%, compared to 2.5% for the rest of the non‐breeding season. There was no marked increase in the percentage of time spent leg tucking when undergoing molt (Appendix S1), consistent with observations of captive puffins (M. Huwiler, Tierpark Bern, pers. comm).

**TABLE 2:** Flightless molt periods identified in the four dual‐equipped individuals.

ID	1st molt start	1st molt duration	2nd molt start	2nd molt duration
EJ47625	7th to 16th Sep	36 to 45 days	20th Feb	32 days
EL60569	1st to 9th Sep	54 to 63 days	—	—
EL60573	17th to 19th Sep	33 to 35 days	9th to 14th Feb	32 to 37 days
EL60648	19th to 21st Sep	44 to 46 days	—	—

*Note*: First molt refers to the earliest molt inferred by the molt identification process, and second molt is any later molt identified as occurring later in the non‐breeding season. A range of results is presented for each puffin, as results vary depending on which logger's time series (left or right leg) is used as the basis for the combination of data.

**FIGURE 4:**
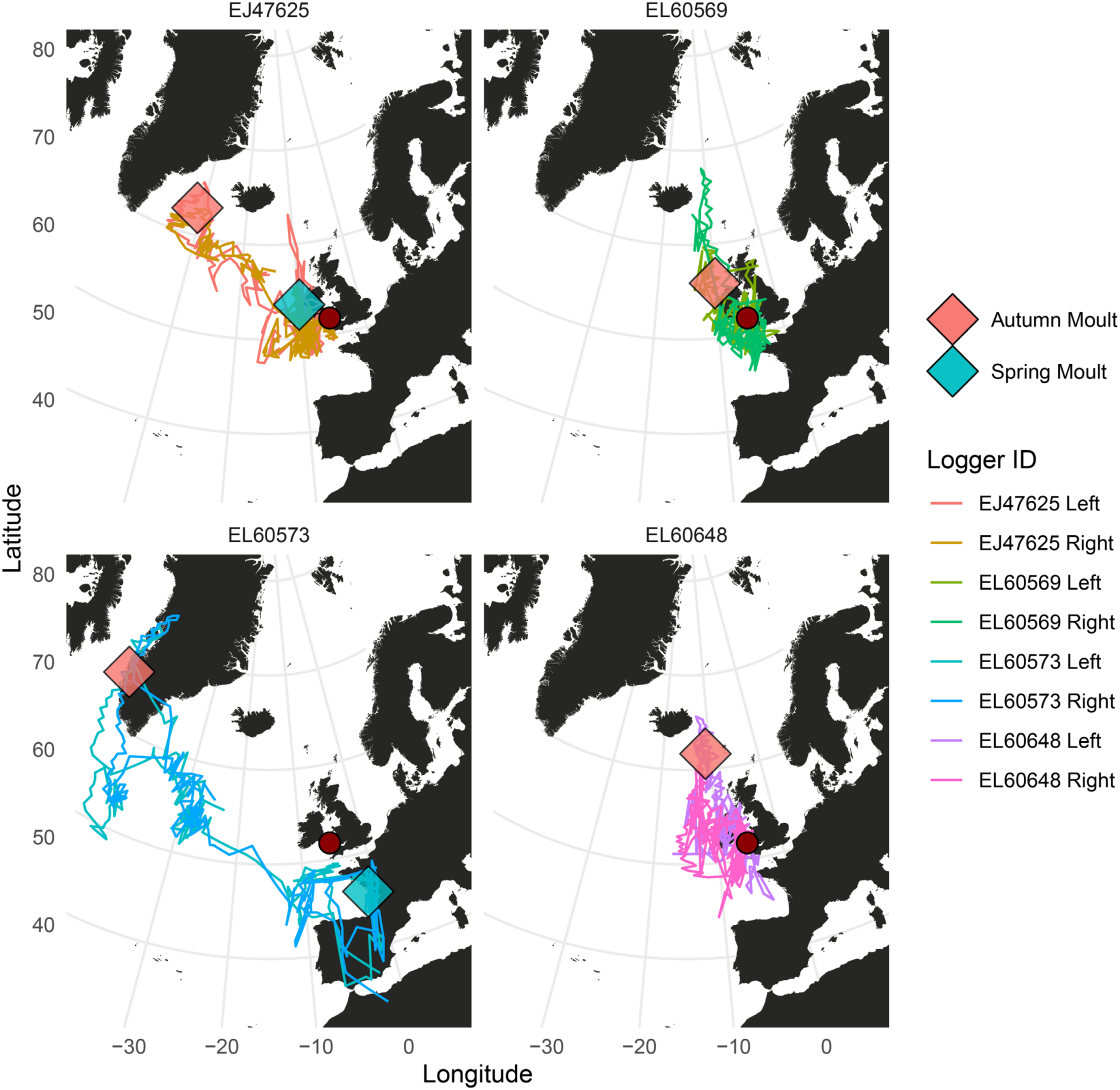
Puffin tracks and associated molt periods. Each plot represents one individual and their mean molt locations, with a track derived from each logger. Geolocator positioning accuracy is reduced around the equinoxes, negatively affecting the latitudinal accuracy of molt locations, which were all inferred to occur around the equinoxes. The red dot corresponds to the colony location, Skomer, Wales. Note that portions of the track apparently crossing land are products of the inaccuracy of geolocator location estimates around the equinoxes and associated smoothing.

All molt periods occurred close to equinox periods when the latitudinal accuracy of light‐level geolocation is greatly reduced. The average position of inferred molt was used to graphically represent molt location (Figure 4), given that puffins are unlikely to move extensively when flightless, and most of the variation in location during molt is almost certainly due to error in location estimates. Both individuals with two inferred molt periods had more extensive migrations than those with one (Table 3), with the autumn flightless molt occurring when they were furthest from their colony and the spring molt when much closer to the colony (Figure 4).

**TABLE 3:** Metrics for each dual‐equipped bird relating to migratory effort and molt periods.

Bird ID	Most distant residency (dist./time)	Inferred molts	Total time in molt
EJ47625	2054 km/49 days	2	77 days
EL60569	728 km/41 days	1	63 days
EL60573	3040 km/42 days	2	72 days
EL60648	1250 km/39 days	1	46 days

*Note*: Residencies were described using Lavielle segmentation of net‐squared displacement from the colony. The number of inferred molt periods and time spent in inferred molt altogether are also shown.

### 3.3 Identifying molt using single‐leg data

Using single‐logger data streams, only one molt period could be successfully identified from the four dual‐equipped individuals, on the left leg of puffin EJ47625. The dates of this, September 16 to October 30, exactly matched the molt period identified using data from both loggers. The percentage of time spent dry in this molt period was 0.4%. No second inferred molt period was detected on the second iteration of the molt identification method, even though this process identified a second molt period for this bird when data from both loggers were combined.

**FIGURE 5:**
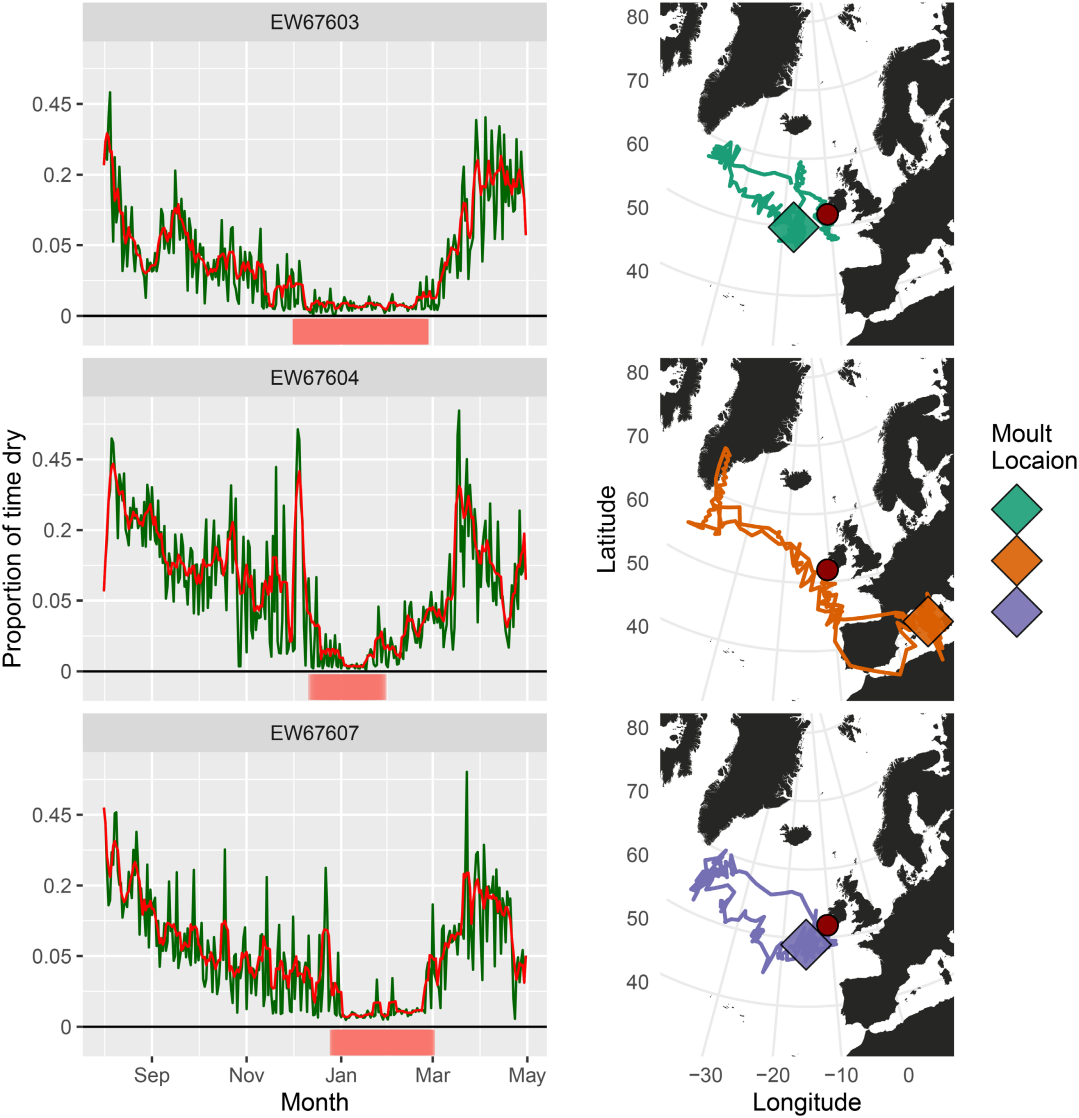
Timing and location of inferred molt of Skellig Michael puffins. On the left, the proportion of time spent dry per day is graphed over the non‐breeding season. The green line represents time spent dry per day (prop_flight_) and the red line is the 5‐day rolling average of this (prop_flight‐5_). The *y*‐axis is square root transformed, with the actual untransformed values displayed. The red bar underneath each plot represents putative molt inferred by the molt identification process. On the right, the corresponding inferred molt location is shown by a diamond shape, overlaid on the migratory path of the individual. The red dot represents Skellig Michael and their breeding colony. Note that portions of the track apparently crossing land are products of the inaccuracy of geolocator location estimates around the equinoxes and associated smoothing.

Of the 40 single‐equipped individuals from Skellig Michael, inferred molt periods were only apparent in three individuals. Periods of sufficiently reduced flight consistent with molt were not detected by our method in any of the other individuals (Appendix S1). These molt periods were 35, 52, and 72 days in duration and occurred in mid‐winter, starting in December or January, and took place either beyond the Irish Atlantic shelf margin (*n* = 2) or in the Mediterranean Sea (*n* = 1) (Figure 5 and Appendix S1). Puffins from Skellig Michael showed reduced leg‐tucking activity around mid‐winter, especially for puffins tagged in 2020, which corresponds with the molt periods inferred for this group (Appendix S1). This suggests that molt is more easily identifiable at this time of year due to an apparent reduction in leg‐tucking behavior, so the temporal distribution of molt inferred here is likely biased toward this period. These three individuals all moved approximately 2000 km from the colony to their furthest point of residency (Appendix S1), comparable to one of the Skomer birds that was inferred to have completed two molts.

## 4 DISCUSSION

### 4.1 Methodology

To identify molt in puffins, we have developed a new method combining multiple data streams from geolocator loggers to identify year‐round behaviors of seabirds more accurately. While this seems to work reasonably well for dual‐equipped individuals, it also highlights some shortcomings of using standard single‐deployment geolocators to identify fine‐scale behaviors. Halpin et al. (2021) show how species' behavior can unpredictably influence location estimates using light‐level geolocators. Leg tucking in alcids presents a similar problem for the interpretation of behaviors from saltwater immersion loggers on the same devices (Fayet et al., 2017; Linnebjerg et al., 2014). We provide a method to partially correct this behavioral classification issue using concurrent light and immersion data. The limitations of this partial correction are reflected in the low success rate of molt inference in single‐logger birds. Because puffins and some other alcids spend very little time in flight even when not undergoing molt (see Section 3, Dunn et al., 2020), flightless molt is impossible to identify without relatively accurate behavioral data, and the few individuals for which molt periods were detected using a single logger are likely biased toward times of the year when leg‐tucking behavior is less prevalent. Despite these limitations, our methods provide new insights into the behavior and life‐history traits of a threatened species and improve our knowledge of the timing and location of a highly vulnerable period in the puffin's annual cycle.

Over the last 10–15 years, hundreds of alcids have been tagged with a single geolocator throughout their biogeographic range (Fayet et al., 2017; Reiertsen et al., 2021), but our method does not have the power to identify molt in a sufficient proportion of individuals to robustly investigate population‐wide patterns. More complex methods, for instance, using machine learning to identify flightless stopovers (Guilford et al., 2009), usually require large amounts of pre‐assigned training data to confidently infer behavior, but may even then be liable to misclassification due to the individual‐ or colony‐level differences in behavior (Bennison et al., 2018). Finer‐resolution data, such as from accelerometers, would allow us to identify flight with much more confidence (e.g., Patterson et al., 2019). GPS loggers would record far more accurate locations, potentially allowing us to identify imposed residency due to flightless molt. To date, none of these alternative devices are small or efficient enough for year‐round deployment on puffins. Geolocators that record temperature can also be used to help correct for leg tucking (Dunn et al., 2020; Elliott & Gaston, 2014), although, like the light‐based corrections used in this study, temperature‐based corrections do not fully capture all instances of leg tucking. A ventrally mounted immersion switch would provide a truer representation of flight/non‐flight behavior. Despite being light enough for long‐term deployment, current techniques to mount these devices long term on the body instead of on a leg ring have been shown to negatively impact the bird's performance (Lameris et al., 2018). For now, dual‐equipped geolocators are probably the most viable method to investigate the flightless molt of puffins and other alcids. As technology improves and devices become smaller, the combined weight of two loggers will have less impact on an animal. Detecting molt in alcids is still contingent on behavior differing at this stage compared to the rest of the non‐breeding season, but results from this study would suggest that is in fact the case.

Stable isotope analysis of feathers sampled during the breeding season may be used to coarsely gauge the location of the most recent primary molt (e.g., St John Glew et al., 2018) and to validate geolocator‐based findings, as the tip of the feather will have similar isotopic properties to the oceanic area in which it was formed even if it is sampled several months later. To complement this, a relatively accurate geolocator‐informed molt timing and location tell us where and when flight feathers were formed, allowing us to analyze the trophic position of food consumed during feather formation using stable isotope analysis (St John Glew et al., 2019). A better understanding of the timing and location of molt may also provide information on the prevalence of toxic chemicals in marine food webs where feathers are being developed by looking at chemical composition of these feathers (Fort et al., 2016).

### 4.2 Biological findings

We have shown that the flightless molt strategy of breeding puffins varies markedly between individuals, and possibly colonies, despite usually being a fixed life‐history trait within migratory bird species (Barta et al., 2008). We also found evidence that some individuals may undergo flightless molt twice in a non‐breeding season, with this strategy possibly tied to more extensive migrations, although this relationship is based on a very small sample size. Puffins spend very little time in flight in the non‐breeding season altogether, so caution is advised when interpreting these prolonged periods of little to no flight as being obligatory due to flight feather molt. However, the duration and timing of these flightless periods are consistent with previous estimates of molt in this species, and if this is the case, our results provide the first evidence for two flightless molt periods per year in a wild volant bird species (Beltran et al., 2018).

The exploration–refinement hypothesis (Guilford et al., 2011) suggests the development over time of a fixed migration strategy that exploits predictable prey availability in space and time, leading to inter‐individual variation (e.g., Harris, Wanless, et al., 2015). More extensive migration may allow puffins to exploit reliable food resources (Jessopp et al., 2013), especially during molt when their diving abilities are likely compromised (Bridge, 2004).

Prolonged flight during migratory phases may lead to accelerated feather wear and reduced flight efficiency for a bird with an already high wing loading (Greenewalt, 1975; Navarro & González‐Solís, 2007), whose burrow‐nesting habits probably cause flight feather wear during the breeding season. Increased energy requirements for long‐distance migrants also necessitate increased foraging effort and dive rates (Fayet et al., 2016), potentially causing further wear in wing feathers. For several reasons, puffins may require two molts in one non‐breeding season to maintain flight feather condition and retain flight efficiency (Barta et al., 2008). It may be that one or both molts are incomplete, allowing the puffins to remain partially volant. Small alcids in the genus *Aethia* forego synchronous molt, instead staging the replacement of primary flight feathers, allowing them to continue flying throughout molt (e.g., Bond et al., 2013). Some storm‐wrecked puffins have shown evidence of a similar partial primary molt (M. Harris, unpublished data), although this has only been observed in a vanishingly small proportion of a very large sample of recovered birds, so is likely an anomaly or due to poor health. Biannual synchronous flightless molts have been observed in captive juvenile puffins (Swennen, 1977; M. Huwiler, Tierpark Bern, pers. comm.), and while it is uncertain how these observations relate to wild breeding adults (Thompson & Kitaysky, 2004), it does highlight that this strategy is physiologically possible. In contrast, the two Skomer individuals that stayed closer to the colony (<1500 km) during the non‐breeding season clearly underwent a single flightless molt in autumn, not long after the summer breeding season. Flight feather molt is energetically demanding (Guillemette et al., 2007) and reduces foraging efficiency (Bridge, 2004), so there are potential advantages in strategies that forego a second flight feather molt where possible. A trade‐off likely exists between the energy required to undergo long‐distance migration to highly productive areas, potentially necessitating two flightless molts, versus reduced migration effort and a single flightless molt in an area where feeding conditions may be poorer. Molt strategy in puffins could be dichotomously (biannual vs. annual molt) associated with high versus low energy intake and expenditure, reflected in the activity budgets of long‐ and short‐distance migrants (Fayet et al., 2017).

Previous studies, based on birds recovered dead rather than those from birds equipped with loggers that survived the non‐breeding season, described an early/late bimodal distribution of puffin molt timings in the North Sea and around the Faroes Islands, with peaks in October and March (Harris et al., 2014). This timing largely agrees with our findings from Skomer individuals. It may be that dead birds identified as molting in March were going through a second molt. Harris et al. (2022) found that almost all birds found wrecked on the East coast of Britain after storms in November and December 2021 had already undergone primary molt, which may reinforce the idea that primary molt in February/March may be a second occurrence. However, many more suitable tracking data, e.g., from dual‐equipped puffins, would be required to suggest this with any confidence. The inferred molt of three Skellig Michael individuals occurred once, from December to February, with no evidence that this followed an earlier post‐breeding flightless molt, although again, this is based on a small sample where molt could be resolved from single‐logger data streams. Similar molt timings were observed by Anker‐Nilssen et al. (2018), who reported that most puffins found following storm wrecks on the coast of Norway in February/March 2016, likely originating from colonies on the East coast of the UK, were in the latter stages of molt and had only recently become volant. Birds found dead are more likely to have been wintering relatively close to land, and so may not provide an unbiased sample of the wider population (Fayet et al., 2017). It is also possible that storms disproportionately affect molting puffins that cannot fly to escape storm tracks, with reduced foraging efficiency during molt further compromised by storm conditions (Clairbaux et al., 2021). This does not seem to universally be the case, with a high proportion of molting birds found in one wreck on the Norwegian coast (Anker‐Nilssen et al., 2018) and a low proportion in another in the Bay of Biscay (Morley et al., 2017), despite both wrecks occurring at a similar time of year.

## 5 CONCLUSIONS

While limited to a small sample size, the results of this study markedly advance our understanding of a vulnerable period in the non‐breeding season of a threatened species. We raise the intriguing possibility that puffins have a unique biannual flightless molt, leaving them flightless for 60–80 days over the course of a single non‐breeding season, accounting for 20%–30% of that total period. This highly unusual strategy may be tied to migration effort, although this is speculative due to our limited sample size. However, we have established a freely available workflow to further analyze dual‐equipped seabirds to improve behavior classification using geolocators. We hope this will promote further research on the flightless molt of puffins and other alcids. At this stage in their annual cycle, they are particularly vulnerable to negative impacts from reduced prey availability, surface pollution, and increased storm prevalence.

## AUTHOR CONTRIBUTIONS

**Jamie Hendrick Darby:** Conceptualization (equal); data curation (equal); formal analysis (lead); investigation (equal); methodology (equal); validation (equal); visualization (lead); writing – original draft (lead); writing – review and editing (equal). **Mike P. Harris:** Conceptualization (equal); investigation (equal); methodology (equal); supervision (equal); visualization (equal); writing – review and editing (equal). **Sarah Wanless:** Conceptualization (equal); investigation (equal); methodology (equal); supervision (equal); writing – review and editing (equal). **John L. Quinn:** Conceptualization (equal); funding acquisition (equal); investigation (equal); methodology (equal); supervision (equal); writing – review and editing (equal). **Vegard Sandøy Bråthen:** Conceptualization (equal); data curation (equal); formal analysis (equal); methodology (equal); writing – review and editing (equal). **Annette L. Fayet:** Conceptualization (equal); investigation (equal); methodology (equal); writing – review and editing (equal). **Manon Clairbaux:** Formal analysis (equal); investigation (equal); methodology (equal); writing – review and editing (equal). **Tom Hart:** Conceptualization (equal); formal analysis (equal); methodology (equal); writing – review and editing (equal). **Tim Guilford:** Conceptualization (equal); data curation (equal); investigation (equal); methodology (equal); writing – review and editing (equal). **Robin Freeman:** Data curation (equal); investigation (equal); writing – review and editing (equal). **Mark John Jessopp:** Conceptualization (equal); formal analysis (equal); investigation (equal); methodology (equal); project administration (lead); supervision (lead); writing – review and editing (equal).

## CONFLICT OF INTEREST

The authors declare no conflict of interest.

### OPEN RESEARCH BADGES

This article has earned Open Data and Open Materials badges. Data and materials are available at: All data used for this publication are available from the Birdlife Seabird Tracking Database (http://seabirdtracking.org/mapper), studies number 1924 and 1926.

## Supporting information


Appendix S1.

## Data Availability

All data used for this publication are available from the Birdlife Seabird Tracking Database (http://seabirdtracking.org/mapper), studies number 1924 and 1926. Data from Skellig Michael are also available as a subset of the SEATRACK database (http://seatrack.seapop.no/map). All code is freely available at github.com/JamieHDarby/gls_puffin_moult.
