# Supplementary material for: A new biologging approach reveals unique flightless molt strategies of Atlantic puffins
Source: Ecol Evol. 2022 Dec 13;12(12):e9579. doi: 10.1002/ece3.9579 (PMC9745502; doi:10.1002/ece3.9579)
Supplement: Supplementary file 1 — Appendix S1. [file ECE3-12-e9579-s001.docx]

**Flightless moult in Atlantic puffins: insights from biologging devices**

**Supplementary material**

**Variation in tucking behaviour**

Two puffins (EJ47625 & EL60569) spent considerably more time tucking their right leg than their left, especially before midwinter, whereas the other two tucked both legs a similar amount throughout the year. One puffin (EL60658) also spent much less of its time tucking than the other three (figure S1).


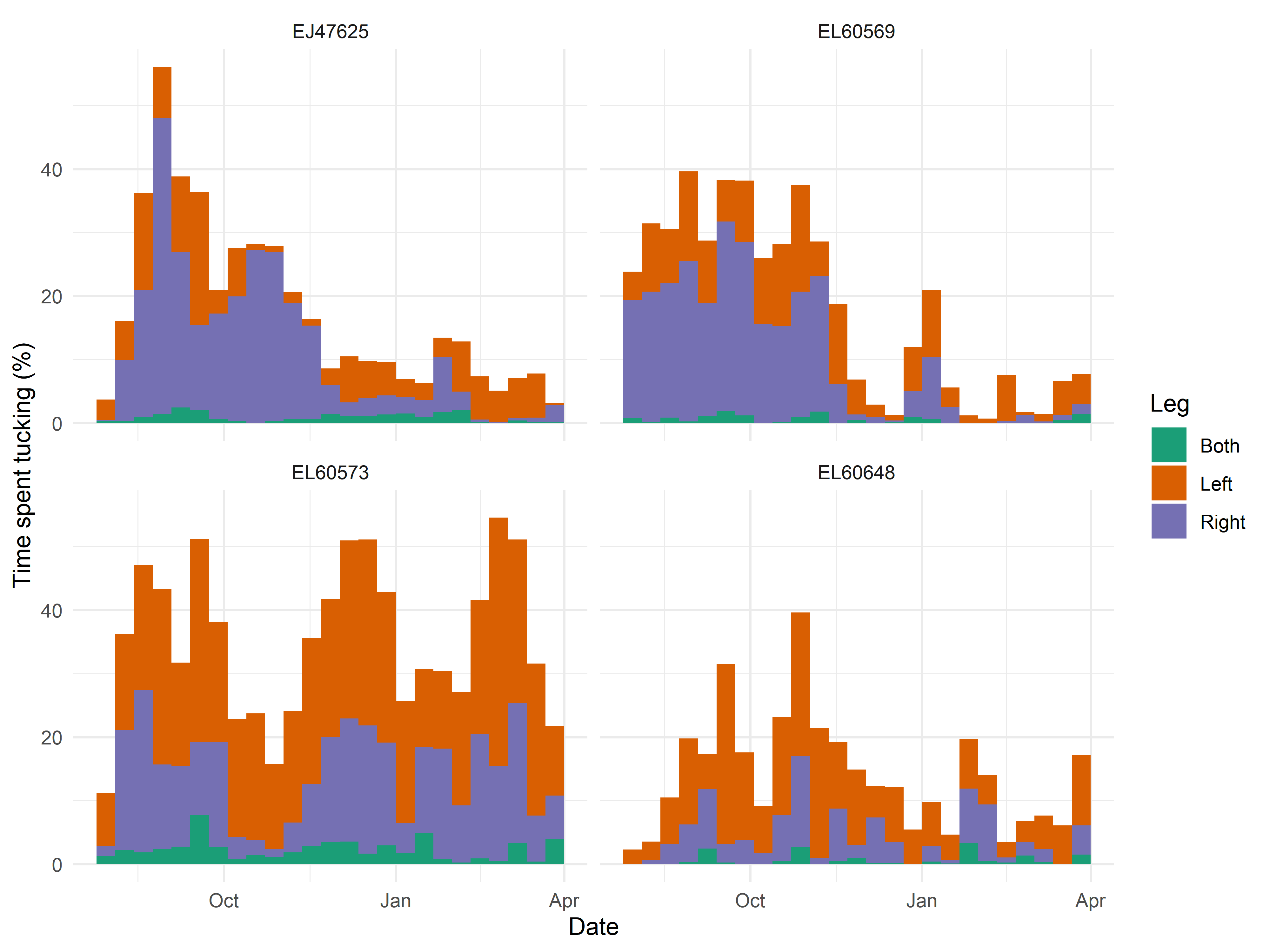


Figure S1: Percentage of daylight time spent leg-tucking, informed by raw light data and predictions. Tucking events are grouped by leg. Each of the 4 plots represent a different individual.

**Tucking while moulting**

No increase in amount of time spent tucking was observed during inferred moult in the dual tagged birds (figure S2). We had hypothesised that puffins may spend more time tucking during moult, due to prolonged time spent resting while flightless, but this does not appear to be the case. Further information from captive puffins reinforces that tucking behaviour doesn’t become more frequent during moult (M. Huwiler 2022, TierPark Bern, pers. comms.), and in contrast, their appetite increases at the onset of moult (D. Dial 2022, National Aquarium USA, pers. comms.), which may result in less time spent leg-tucking in the wild.

**
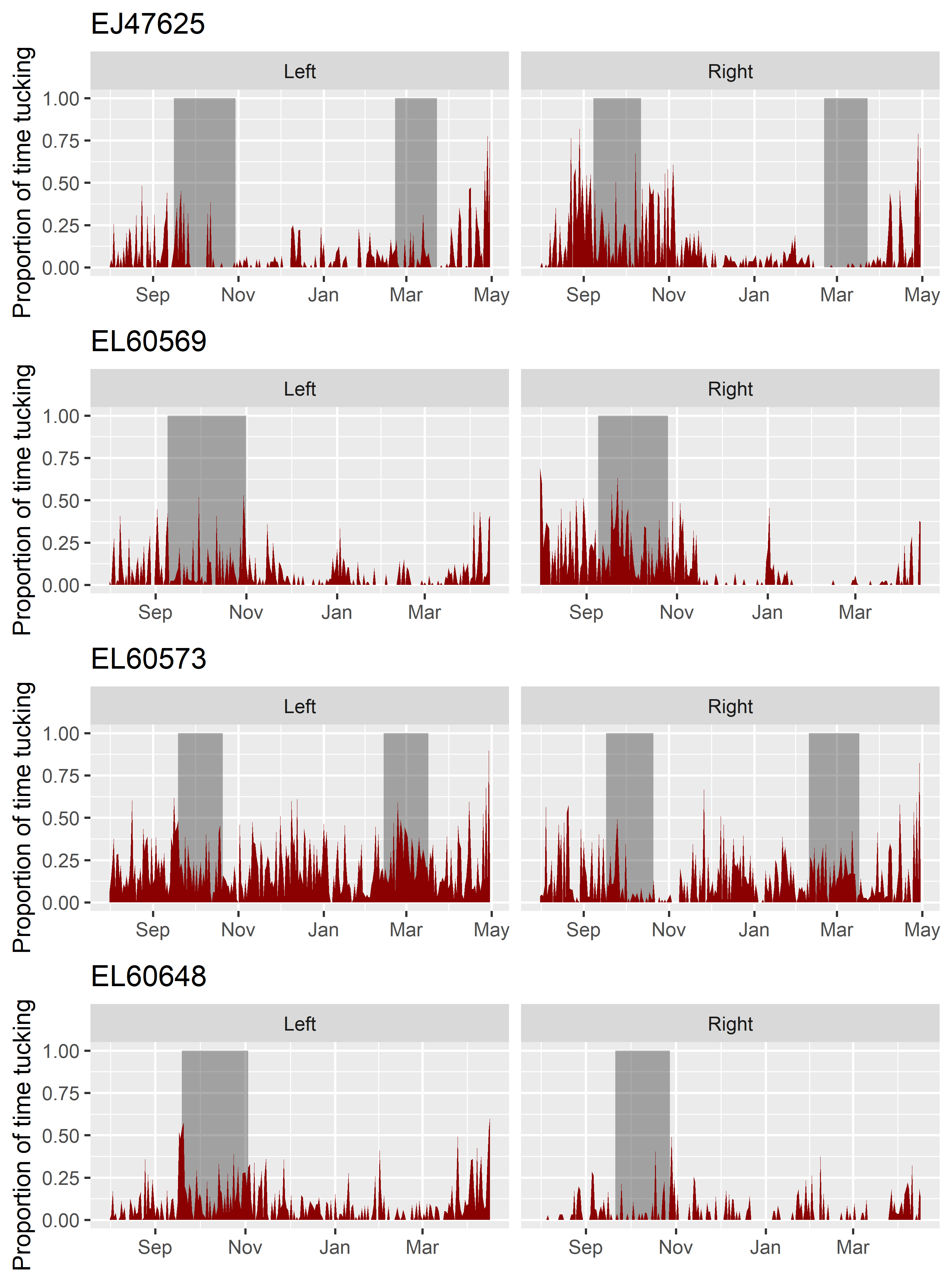
**

Figure S2: Graph showing the amount of time each dual-tagged puffin spent tucking each leg per day over the non-breeding season (dark red), with moult periods overlaid (grey shading).

**Tucking behaviour of Skellig Michael puffins**

Tucking behaviour was much more prevalent in autumn than in winter in Skellig Michael puffins (figure S3). The peak in “tucking” behaviour around the end of April/start of May possibly corresponds to the first visits to the study colony and burrow nests, where the light-logger will be obscured underground during daylight hours. The reduced leg-tucking activity around midwinter, especially for puffins tagged in 2020, corresponds with the moult periods inferred for this group. This suggests that moult is more easily identifiable at this time of year due to an apparent reduction in leg-tucking behaviour, so the temporal distribution of moult inferred here is likely biased towards this period.

**
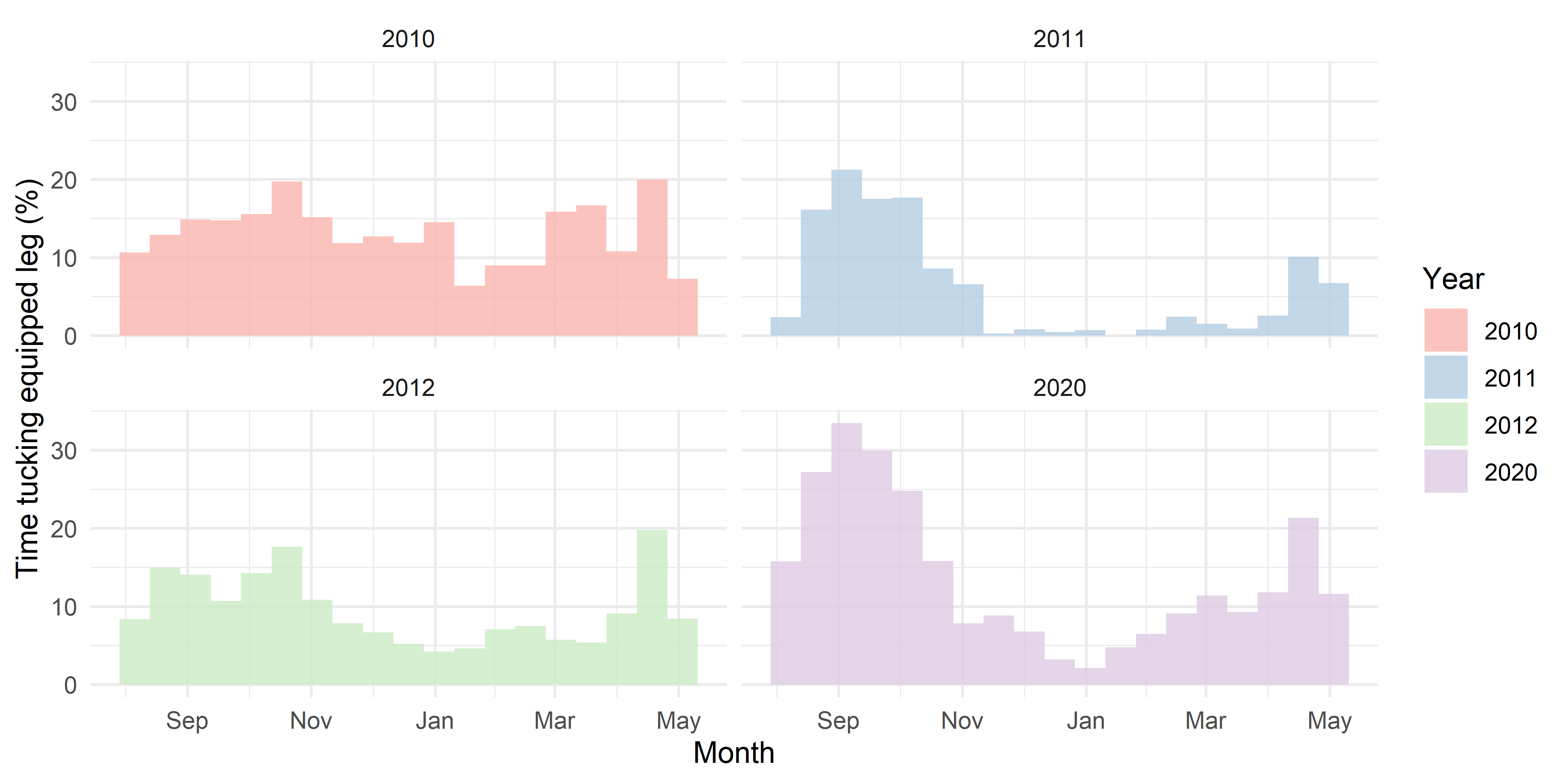
**

Figure S3: Proportion of time spent leg-tucking per daylight interval (solar angle > -3), informed by raw light data and GAM predictions. Tucking events are grouped by year and pooled across all Skellig Michael study individuals. Year refers the non-breeding season beginning August of that year and ending in May of the next.

**Moult metrics for Skellig Michael puffins**

Table S1: Date, duration, and coarse location of inferred moult of Skellig Michael puffins.

| ID | Moult start | Moult duration | Location |
| --- | --- | --- | --- |
| EW67603 | 9^th^ Dec | 72 days | Atlantic |
| EW67604 | 19^th^ Dec | 35 days | Mediterranean |
| EW67607 | 2^nd^ Jan | 52 days | Atlantic |

**Migration metrics for Skellig Michael puffins**

Table 1: Distance and time spent at the furthest residency along the migratory track. Periods of residency are inferred by Lavielle segmentation. See methods in the main text for further details.

| ID | Most distant residency (dist / time) |
| --- | --- |
| EW67603 | 1820km / 92 days |
| EW67604 | 2073km / 71 days |
| EW67607 | 1900km / 104 days |

**Failed moult identification for Skellig Michael puffins**

**
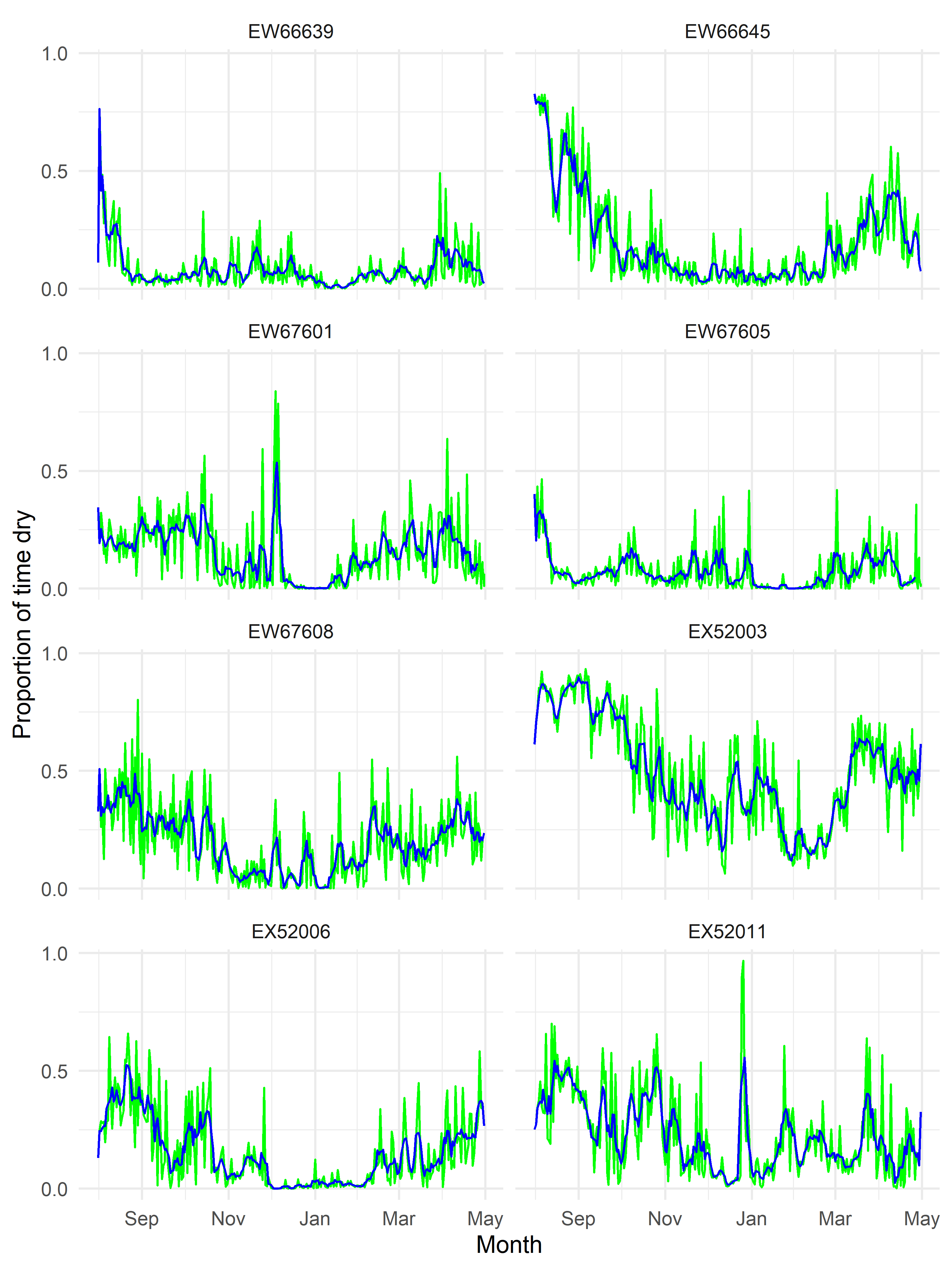
**

Figure S4: Time series showing the proportion of flight for day (green line) and 5-day rolling average of this (blue line) of 8 Skellig Michael puffins after tucking correction. Our moult identification did not infer moult periods in any of these time series.

Figure S4 shows time series from single-equipped geolocators from 8 puffins for which no moult was inferred by our method. Though periods of reduced flight are visually apparent in certain cases (see EW67601, EW67605), they did not meet the criteria for classification as moult by our method. This is due to the rolling average of time spent dry not remaining below 1% per day for a sufficient period. According to the results from dual-equipped data, these periods may represent periods of residency during which the puffin did not fly extensively but was not obligatorily flightless. Tucking is shown to be less prevalent during these periods, which mostly occur in midwinter (figure S3). Shorter daylengths at this time may necessitate increased foraging effort in puffins for the limited daylit time available, reducing time available for leg-tucking/resting.
